# Application of Controlled-Release Urea Enhances Grain Yield and Nitrogen Use Efficiency in Irrigated Rice in the Yangtze River Basin, China

**DOI:** 10.3389/fpls.2018.00999

**Published:** 2018-07-19

**Authors:** Li Wang, Cheng Xue, Xia Pan, Fang Chen, Yi Liu

**Affiliations:** ^1^Key Laboratory of Aquatic Botany and Watershed Ecology, Wuhan Botanical Garden, Chinese Academy of Sciences, Wuhan, China; ^2^College of Resources and Environment Science, Hebei Agricultural University, Baoding, China; ^3^China Program, International Plant Nutrition Institute, Wuhan, China

**Keywords:** controlled-release urea, nitrogen use efficiency, leaf senescence, dry matter and nitrogen remobilization, post-anthesis nitrogen uptake

## Abstract

The use of controlled-release urea (CRU) has been recommended over that of conventional urea to improve rice grain yield and nitrogen use efficiency. However, the underlying agronomical and physiological mechanisms need to be better understood. In this study, field trials over four site-years, and a big container experiment were carried out to explore CRU effects on rice yield and NUE, with the main aims to identify the key yield components contributing to the superior rice yield with CRU use, and to evaluate differences in dry matter, nitrogen (N) accumulation, translocation and yield formation with different N fertilizer practices. Four N treatments were investigated: control with 0 kg N ha^−1^ (CK), farmers' fertilizer practice (FFP) with 150 kg N ha^−1^ as urea basal application, modified fertilizer practice (MFP) with 150 kg N ha^−1^ as split urea application (40% at transplanting, 30% at tillering and 30% at the panicle stages), and CRU treatment with 150 kg N ha^−1^ as CRU basal application. Results showed that the CRU increased rice yields by 10.8 and 5.6% over FFP and MFP, respectively. The N recovery efficiency and N agronomic efficiency for CRU were significantly higher than that obtained from MFP and FFP treatments. The analysis of yield components revealed that the higher grain yields using CRU were accounted for mainly by increased panicle and spikelet numbers per m^2^, which resulted from higher N uptake. In addition, results from the container experiment with comparable experimental design to field trials illustrated that both post-anthesis dry matter production and translocation were critical for high grain yields using CRU, while the former seemed more important. Relative to MFP and FFP, CRU maintained higher flag leaf SPAD and photosynthetic rate, as well as higher root oxidation activity (ROA) and N uptake during grain filling. Furthermore, CRU increased the activities of key enzymes involved in N assimilation in flag leaves, including GS, GOGAT, and NR. CRU effects on such underground and aboveground processes were proposed to contribute to high rice yield.

## Introduction

Rice (*Oryza sativa* L.) is the major cereal crop and staple food for more than half the world's population (Borah and Baruah, 2016). By the year 2030, rice production in China should increase 14% (relative to 2010) to meet the food requirement from the growing population (Cheng et al., 2007). Increasing use of chemical nitrogen (N) fertilizer since 1970 has greatly enhanced rice yield, but N over-fertilization has become widespread (Huang and Tang, 2010; Chen J. et al., 2011). Such over-fertilization has not only lowered N use efficiency (NUE), but also increased N losses from paddy field soil to the environment through different pathways, contributing to the reduction of air, water and soil quality (Wang et al., 2016; Xia et al., 2016). A mounting concern is therefore how to increase grain yield while at the same time reducing the environmental impact of intensive agriculture (Chen X. P. et al., 2011).

Much effort has been put into improving crop management technologies to increase fertilizer NUE while reducing N losses (Peng et al., 2010; Chen et al., 2015; Hofmeier et al., 2015). Using computerized decision support systems, chlorophyll meters (SPAD), and leaf color charts, the International Rice Research Institute has developed site-specific N management techniques, such as fixed-time adjustable dose N management and real-time N management (Yadvinder et al., 2007; Huang et al., 2008; Peng et al., 2010). These have been proven to be effective in achieving high yield and N use efficiency with reduced N input in irrigated rice systems. In addition, crop management based on multi-split topdressing have been recommended as one of alternative approaches to increase simultaneously the grain yield, NUEs and production profit with reduced N application in the Yangtze River Basin in China (Chen et al., 2015). However, some of these approaches require extensive technical support, based on monitoring tools or involve a large amount of labor and working in muddy fields. Since farm sizes in China usually being very small, and a growing number of young farmers migrating from rural to urban areas for jobs (Levine et al., 2008), the dissemination of such technologies has been limited. A simpler and more convenient N fertilizer management is hence required.

Several studies have demonstrated the effects of controlled release N fertilizer in enhancing rice yield and NUE (Ye et al., 2013; Geng et al., 2015; Zheng et al., 2017). Moreover, the advantage of controlled-release urea (CRU) is that it can be applied as a single basal dose, making it convenient for farmers to implement. Theoretically, the cumulative N release of CRU follows a “S” shape curve over time, which could provide better synchronization with rice N demands than traditional fertilizers. Particularly, certain CRUs could provide a sustained N supply to rice crops through prolonged N release, which is crucial for increasing N uptake at late growth stages of rice and thus, grain yield (Yang et al., 2012; Geng et al., 2015). However, it is worth noting that there is substantial variability in the reported benefits of CRU in increasing crop yield and NUE (Golden et al., 2009; Yang et al., 2012; Li et al., 2018). Differences in coating materials as well as in the environmental conditions of given regions may lead to variations in N release characteristics and the synchronization with crops' demands. However, previous studies evaluating CRU effects over multiple site-years are rare, despite their importance and necessity.

Although CRU has been recommended as an effective means to increase rice yield, the underlying agronomical and physiological mechanisms are not well documented. Rice yield is determined by yield components, including effective panicle number (PN), number of spikelet per panicle (SPP), spikelet number (SN) per m^2^, grain weight (GW), and grain filling (GF) percentage. Crop management based on multi-split topdressing is known to influence rice yield through regulation of yield components such as PN and SN, with SN being considered as the main factor determining yield (Chen et al., 2015). In contrast, the grain weight of rice is a very stable varietal character, as spikelet size is rigidly controlled by hull size under most conditions (Sui et al., 2013; Wu et al., 2013). Despite the advances in our understanding of N influence on rice yield components, the CRU effect still needs to be better understood.

From another perspective, grain yield could be derived from two sources, i.e., post-anthesis biomass production and translocation of biomass accumulated pre-anthesis. Previous work has emphasized the importance of high crop biomass accumulation at anthesis, which could allow greater remobilization of resources to the developing grain (Pal et al., 2017). It has been reported that up to 83% of straw non-structural carbohydrate and 68% of straw N accumulated pre-anthesis could be translocated to grains during ripening, depending on genotypes and growing conditions (Yang et al., 2001; Pal et al., 2017). On the other hand, evidence is now mounting that dry matter (DM) production post-anthesis may be a major contributor to grain yield in rice (Wu et al., 2013; Mahajan and Chauhan, 2016; Pal et al., 2017). So far, there is a dearth of reports on the effects of CRU fertilizers on these key processes. Specifically, leaves play two important roles during the reproductive stage, e.g., they are the major source organ for grain filling, and act as the main photosynthesis organ vital for DM production post-anthesis. Delaying leaf senescence and maintaining relatively high rates of photosynthesis are preferable for high grain yield, which could be regulated by soil N supply in the later stages (Zhang et al., 2011, 2013). Several studies, as well as our own investigation, have indicated the role of certain CRUs in delaying leaf senescence and enhancing photosynthesis post-anthesis (Yang et al., 2012; Geng et al., 2016). It could be hypothesized that the prolonged N release from CRU facilitates post-anthesis N uptake, leading to delayed leaf senescence, superior dry matter production and increased rice yield.

Recently, a new type of environment-friendly CRU (polyurethane-coated CRU containing 44% N, Agrium Inc, 2016) has been introduced to farmers and used in at least 10 provinces in China, due to its low cost and predictable release pattern. Our previous work has proven its role in providing a better synchronization between soil N supply and rice demand than traditional N fertilizers, reducing N losses through ammonia volatilization and surface runoff, and thus enhancing rice yield and N use efficiency (Li et al., 2017, 2018). However, it leaves open the question of what are the critical physiological factors and agronomical traits that determine the high yield performance in irrigated rice cropping systems using CRU. To fill this gap, field trials over four site-years as well as a big container experiment were carried out in this study. The aims were as follows: (1) to evaluate the effects of CRU application on rice grain yield and NUE in comparison with traditional urea; (2) to identify the main yield components determining yield performance, and (3) to analyze the regulation of the source-sink relationship during yield formation and the contributions of key processes before and after anthesis.

## Materials and methods

### Field experiments

Field experiments were carried out in Honghu City, Hubei province (30.05°N, 113.73°E) and in Nanchang County, Jiangxi province (28.37°N, 115.92°E) in 2009 and 2010. These experimental sites were within the major rice-production areas in the Yangtze River Basin with a subtropical climate. Monthly precipitation, mean temperature and hours of sunlight are given in Table 1. Soils in the paddy fields were classified as Entisol (Soil Survey Staff, 1999). The initial soil properties of the plow layer (0–20 cm) are shown in Table 2.

**Table 1:** Monthly precipitation, hours of sunlight and mean temperature during the rice growing season.

**Year**	**Location**	**Experiment**	**Precipitation (mm)**	**Mean temperature (^°^C)**	**Hours of sunlight (h)**
2009	Honghu	Field trial	96.4	27.0	172.7
2009	Nanchang	Field trial	115.5	27.1	217.0
2010	Honghu	Field trial	146.0	26.6	159.1
2010	Nanchang	Field trial	338.0	26.1	149.3
2016	Wuhan	Container trial	301.9	27.1	185.0

**Table 2:** Physical and chemical properties of experimental soils.

**Location**	**pH**	**Organic matter (g kg^−1^)**	**Alkali-N (mg kg^−1^)**	**Olsen-P (mg kg^−1^)**	**Available K (mg kg^−1^)**
Honghu	6.60	23.0	145	26.9	107.5
Nanchang	5.32	31.2	194	26.4	198.0
Wuhan	5.71	15.8	87	21.2	127.2

Field experiments were carried out with a randomized complete block design in triplicates of four treatments, namely: (1) control with 0 kg N ha^−1^ (CK), (2) local farmers' fertilizer practice (FFP), with 150 kg N ha^−1^ as a basal application in urea, (3) modified fertilizer practice (MFP), with 60, 45 and 45 kg N ha^−1^ in urea applied at the basal, tillering and panicle initiation stages, respectively, and (4) CRU application, with 150 kg N ha^−1^ as a basal application using CRU. In addition, 90 kg P_2_O_5_ ha^−1^ and 90 kg K_2_O ha^−1^ in the form of calcium magnesium phosphate and potassium chloride were applied for each treatment 1 day before transplanting. The urea used in FFP and MFP treatments was prilled urea containing 46.4% N, provided by SINOFERT, Ltd, and the CRU was polyurethane-coated urea containing 44% N, provided by Agrium Advanced Technologies, Inc. The individual plots (5 m × 5 m) were edged with plastic film inserted to a depth of 30 cm below the soil surface to prevent seepage between adjacent plots.

The rice cultivar used in this study was Liangyou 6326. The previous crop grown at the experimental sites was oilseed rape. Rice seedlings were transplanted at a density of 24 and 21.5 hills m^−2^ (3 plants hill^−1^) in Honghu in 2009 and 2010, respectively, while the planting density in Nanchang was 25.7 hills m^−2^ for both years. A water layer approximately 10 cm deep was maintained during the tillering stage. This was drained, starting at the maximum tillering stage for 10 days and then refilled to a depth of 5 cm when the visible water layer disappeared. Weeds, pests and diseases were well controlled to avoid yield loss.

At maturity, a 5 m^2^ area in the middle of each plot was harvested to measure grain yield and the aboveground biomass. Twelve individual plants were selected randomly to assess the panicles, spikelet number per panicle. Grain filling percentage and 1,000-grain weight were assessed. DM from different plant parts were determined after drying plant samples at 70°C to a constant weight. For analysis of N content, straw and grain samples were ground into powder and then digested with H_2_SO_4_-H_2_O_2_ at 270°C. Total N concentration was measured using a continuous-flow injection analyzer (AA3, Bran and Luebbe, Norderstedt, Germany).

The NUEs of the N recovery efficiency (RE_N_), agronomic use efficiency (AE_N_), internal N use efficiency (IE_N_) and N partial factor productivity (PFP_N_) were calculated using the following formulae:

(1) $$\begin{array}{r} {\text{RE}}_{\text{N}}=\left({\text{T}}_{\text{N}}-{\text{T}}_{0}\right)/{\text{F}}_{\text{N}} \end{array}$$

(2) $$\begin{array}{r} {\text{AE}}_{\text{N}}=\left({\text{G}}_{\text{N}}-{\text{G}}_{0}\right)/{\text{F}}_{\text{N}} \end{array}$$

(3) $$\begin{array}{r} {\text{IE}}_{\text{N}}={\text{G}}_{\text{N}}/{\text{T}}_{\text{N}} \end{array}$$

(4) $$\begin{array}{r} {\text{PFP}}_{\text{N}}={\text{G}}_{\text{N}}/{\text{F}}_{\text{N}} \end{array}$$

where T_N_ and T_0_ are the total N uptake in the plots with and without N fertilizer application, respectively, G_N_ and G_0_ represent grain yields in the corresponding plots, respectively, and F_N_ is the amount of N fertilizer applied.

### Container experiment

A container experiment was carried out in Wuhan Botanical Garden, Chinese Academy of Sciences in 2016, an area characterized by a monthly precipitation of 301.9 mm, sunlight hours of 185.0, and a mean temperature of 27.1°C during rice growing season (Table 1).

At the start of the experiment, big plastic containers (130 L, 50 cm in diameter and 70 cm in height) were filled with 15 cm of sand in thickness at the bottom, and above that with 100 kg of air-dried soil containing 15.8 g kg^−1^ organic matter, 87 mg kg^−1^ alkali-N, 21.2 mg kg^−1^ Olsen-P, and 127.2 mg kg^−1^ available K with a pH of 5.71 (Table 2). Two weeks before transplanting, a water layer approximately 10 cm in depth was maintained in each container. Water management was similar to that of the field experiments.

Four N fertilizer treatments, CK (N0), FFP (2.94 g N container^−1^ in urea applied as a basal dose), MFP (1.18, 0.88, and 0.88 g N container^−1^ applied at basal, tillering and panicle initiation stages, respectively) and CRU (2.94 g N container^−1^ in CRU applied as a basal dose) were set up with 6 replicates. Apart from N fertilizer, each container received 1.47 g P_2_O_5_ and 2.36 g K_2_O in the form of calcium magnesium phosphate and potassium chloride applied 1 day before transplanting. Rice seedlings (cv. Liangyou 6326) were transplanted with a density of 4 hills per container (3 plants hill^−1^). Over the whole growing season, rice plants were kept under natural conditions outside. Necessary measures were taken to control weeds, pests and diseases.

To determine DM and N translocation during grain filling, three replicates from each treatment were harvested at anthesis and physiological maturity, respectively. Plant samples were separated into culms, leaves and panicles at anthesis, and into culms, leaves, chaff and grains at maturity. All the samples were oven-dried at 70°C to a constant weight. Subsamples were ground to powder for N analysis following the method described in the field experiments section.

Post-anthesis DM and N accumulation were calculated as the difference between the aboveground accumulation at maturity and at anthesis (Pal et al., 2017). Assuming all of the DM and N losses from vegetative plant parts were translocated to the developing grains, the DM translocation (DMT) and N translocation (NT) during grain filling were estimated, as described by Papakosta and Gagianas (1991) as:

(5) $$\begin{array}{r} \text{DMT}=\text{D}{\text{M}}_{\text{a}}-\left(\text{D}{\text{M}}_{\text{leaf},\text{m}}+\text{D}{\text{M}}_{\text{culm},\text{m}}+\text{D}{\text{M}}_{\text{chaff},\text{m}}\right) \end{array}$$

(6) $$\begin{array}{r} \text{NT}=\text{N}{\text{T}}_{\text{a}}-\left(\text{N}{\text{T}}_{\text{leaf},\text{m}}+\text{N}{\text{T}}_{\text{culm},\text{m}}+\text{N}{\text{T}}_{\text{chaff},\text{m}}\right) \end{array}$$

in which DM_a_ is the DM of the aboveground part at anthesis, DM_leaf,m_, DM_culm,m_, and DM_chaff,m_ are the DM of leaves, culm, and chaff at maturity, respectively, NT_a_ is the N content of the aboveground part at anthesis, and NT_leaf,m_, NT_culm,m_ NT_chaff,m_ are the N content of leaves, culms, and chaff at maturity, respectively.

The DM translocation efficiency (DMTE) and N translocation efficiency (NTE) were calculated as:

(7) $$\begin{array}{r} \text{DMTE}=\text{DMT}/\text{D}{\text{M}}_{\text{a}}\;\times \;100\% \end{array}$$

(8) $$\begin{array}{r} \text{NTE}=\text{NT}/\text{N}{\text{T}}_{\text{a}}\;\times \;100\% \end{array}$$

The contribution of pre-anthesis DM remobilization to grain (CDMRG) and N assimilation to grain (CNRG) were calculated as:

(9) $$\begin{array}{r} \text{CDMRG}={\text{DMT}/\text{DM}}_{\text{grain}} \end{array}$$

(10) $$\begin{array}{r} \text{CNRG}={\text{NT}/\text{N}}_{\text{grain}} \end{array}$$

DM_grain_ and N_grain_ are the DM and N content of grain at maturity, respectively.

To monitor the process of leaf senescence during grain filling, the chlorophyll contents of flag leaves were estimated non-destructively using a portable chlorophyll meter (SPAD-502, Minolta, Japan). Four flag leaves per container were measured and three readings per leaf were taken. The measurements were repeated on the same leaves every 7 days. The corresponding photosynthetic rates of flag leaves were measured during morning hours using a portable gas exchange system (LI-6400, LI-COR, Lincoln, NE, USA) with an incoming photosynthetic photon flux density of 1,500 μmol m^−2^ s^−1^, and an ambient CO_2_ concentration of 400 μmol mol^−1^.

To measure root oxidation activity (ROA), three soil cores were taken at three positions in each container between rice hills immediately after photosynthesis measurements at 3 and 17 days after anthesis (DAA). A steel tube (9 cm in diameter, 60 cm in length) was driven into the soil down to 30 cm. The roots in each soil core were carefully rinsed. After combining roots from the soil cores in each container and recording the fresh weight, portions of each root sample were used for the measurement of ROA or root dry weight. The ROA was determined by measuring the oxidation of alpha-naphthylamine (α-NA), as described by Ramasamy et al. (1997). Meanwhile, five flag leaves per container were sampled, frozen in liquid nitrogen and kept at −70°C for further analysis of activities of N metabolism enzymes, including nitrate reductase (NR), glutamine synthetase (GS), glutamine 2-oxoglutarate aminotransferase (GOGAT). In brief, the NR activity was measured following Li (2006), and NR activity is expressed in μmol NO_2_ g^−1^ FW h^−1^, which referred to 1 mol NO_2_ produced in 1 h by 1 g fresh weight of plant samples at 25°C. The GS activity was analyzed by the method as described by Lea et al. (1990), and one unit of GS activity equals the amount of enzyme catalyzing the formation of 1 μmol of γ-glutamyl hydroxamate per minute at 37°C. The GOGAT activity was determined by the method of Singh and Srivastava (1986), and one unit was defined as that reducing 1 μmol nicotinamide adenine dinucleotide (NADH) in reaction mixture per minute at 30°C.

In parallel with the container trials, the N release characteristics of CRU was measured by the burial method (Yang et al., 2012). In brief, the CRU samples (10.0 g) placed in double layered bags (5 × 5 cm, with mesh size of 1.0 mm) were buried to a depth of 10 cm in the soil before rice transplanting. Three bags were randomly collected at each sampling time after 1, 4, 8, 32, 52, 72, and 92 days, until the cumulative amount of N release was above 80%. The bags were cleaned with de-ionized water to remove soil particles, and then the residual N was determined after drying to calculate the N release rate.

### Data analysis

The experimental data were statistically analyzed using SPSS 16.0. Two-way analysis of variance (ANOVA) was applied to test the effects of N treatment, year, site and the interactions between factors. Differences between the treatments were calculated using the Turkey test at 0.05 probability level. Figures were made using SigmaPlot 10.0 software.

## Results

### Field experiments

#### Grain yield and yield components

N treatment had a significant effect on grain yield, as shown in Table 3. The grain yield in the CK plots were consistently the lowest among N treatments. In contrast, FFP had a grain yield 6.4–27.9% higher (*P* < 0.05). MFP further improved grain yield to a range of 7.10–8.02 t ha^−1^, yields that were, remarkably, higher than from FFP in three out of four field experiments (except for Honghu 2009). Grain yield was improved the most by CRU application, i.e., 5.8% higher than MFP on average. Moreover, grain yield was influenced by year, location and their interaction, namely higher in 2009 than 2010 (*P* < 0.05), and higher in Nanchang than in Honghu (*P* < 0.05).

**Table 3:** Grain yield and its components in different fertilizer treatments.

**Year (Y)**	**Location (L)**	**Treatment (T)**	**GY (Mg ha^−1^)**	**PN (m^−2^)**	**SPP (panicle^−1^)**	**SN (m^−2^)**	**GF (%)**	**GW (mg)**	**HI (%)**	**ATB (Mg ha^−1^)**
2009	Honghu	CK	6.74 a	180.0 a	121.0 a	21,628 a	88.6 a	27.2 a	52.2 b	12.9 a
		FFP	7.94 b	247.5 b	130.1 a	32,158 b	87.8 a	27.6 a	46.7 a	16.9 b
		MFP	8.02 b	259.5 b	130.3 a	33,734 b	88.1 a	28.1 a	47.0 a	17.1 b
		CRU	8.22 c	252.5 b	131.6 a	33,163 b	88.3 a	27.7 a	48.2 a	17.4 b
		Mean	7.75	234.9	128.3	30,170	88.2	27.6	48.5	16.1
	Nanchang	CK	6.34 a	176.5 a	135.3 a	23,820 a	85.7 a	26.0 a	50.9 b	12.5 a
		FFP	7.60 b	237.4 b	143.3 a	34,006 b	86.1 a	26.5 a	47.3 a	16.1 b
		MFP	7.99 c	253.1 b	144.5 a	36,596 c	85.8 a	26.1 a	48.8 a	16.3 b
		CRU	9.00 d	272.4 c	144.6 a	39,448 d	85.8 a	26.1 a	49.7 ab	18.2 c
		Mean	7.73	234.9	141.9	33,467	85.9	26.2	49.2	15.8
2010	Honghu	CK	6.23 a	133.0 a	127.0 a	16,801 a	89.6 a	28.6 a	50.4 c	12.4 a
		FFP	6.63 b	146.7 b	150.0 b	21,992 b	91.1 a	28.7 a	45.1 a	14.5 b
		MFP	7.10 c	165.6 c	162.1 b	27,290 c	89.3 a	28.2 a	47.0 b	15.1 b
		CRU	6.91 c	166.5 c	163.6 b	26,884 c	89.7 a	28.4 a	48.6 b	14.5 b
		Mean	6.74	153.2	150.7	23,242	89.9	28.5 a	47.5	14.1
	Nanchang	CK	5.63 a	171.3 a	132.6 a	22,650 a	83.5 a	25.5 a	49.9 c	11.3 a
		FFP	7.20 b	231.3 b	140.5 a	32,444 b	82.9 a	25.7 a	45.8 a	15.7 b
		MFP	7.67 c	246.8 b	140.3 a	34,608 c	83.1 a	25.1 a	47.4 b	16.0 b
		CRU	8.43 d	257.0 b	140.4 a	36,054 d	82.7 a	25.6 a	48.0 b	17.6 c
		Mean	7.23	226.6	138.5	31,439	83.1	25.5	48.0	15.2
ANOVA		T	^***^	^***^	^***^	^***^	ns	ns	^***^	^***^
		Y	^***^	^***^	^***^	^***^	ns	ns	^***^	^**^
		L	^*^	^***^	ns	^***^	^***^	^***^	^*^	ns
		T × Y	ns	^*^	ns	ns	ns	ns	ns	ns
		T × L	^***^	^**^	^*^	^**^	ns	ns	ns	ns
		Y × L	^**^	^***^	^***^	^***^	ns	ns	ns	ns

*Within a column for each location, means followed by the same letters are not significantly different at 5% level according to LSD. GY, grain yield; PN, panicle number; SPP, spikelet number per panicle; SN, spikelet number; GF, grain filling percentage; GW, grain weight; HI, harvest index; ATB, aboveground biomass*.

Panicle number was affected by N treatment, year, location and their interactions (Table 3). CK produced the lowest overall panicle numbers among the treatments. In contrast, FFP increased panicle numbers by 10.3–37.5%. The panicle numbers from MFP were consistently higher (4.8–12.9%) than FFP, but the difference was only statistically significant at Honghu 2010. CRU further increased panicle numbers to levels ranging from 166.5 to 257.0 m^−2^. The mean panicle number at Honghu 2010 was substantially lower than over other three site-years.

CK showed consistently the lowest spikelet numbers. Relative to CK, FFP and MFP significantly increased spikelet numbers by 30.9–48.7%, and 52.8–62.4%, respectively. The spikelet numbers for the CRU treatment were consistently the highest, and were significantly higher than MFP in two out of four trials (at Nanchang 2009 and 2010). However, spikelet numbers per panicle did not differ significantly among N treatments, indicating the highest spikelet numbers per m^−2^ for CRU were mainly attributed to greater panicle numbers per m^−2^ rather than a big panicle size. Moreover, we did not find marked N effects on grain weight and grain filling percentage.

The amount of aboveground total biomass (ATB) varied with N treatments and experimental site-years. Table 3 shows that ATB was the highest in CRU, then MFP and FFP, with the lowest in CK.

CK had a harvest index (HI) ranging from 49.9 to 52.2%. N application significantly reduced HI. In 2009, FFP, MFP, and CRU treatments did not differ in HI. In 2010, CRU and MFP were similar in crop HI, but were significantly higher than that for FFP.

### N uptake and N use efficiency

**Figure 1:**
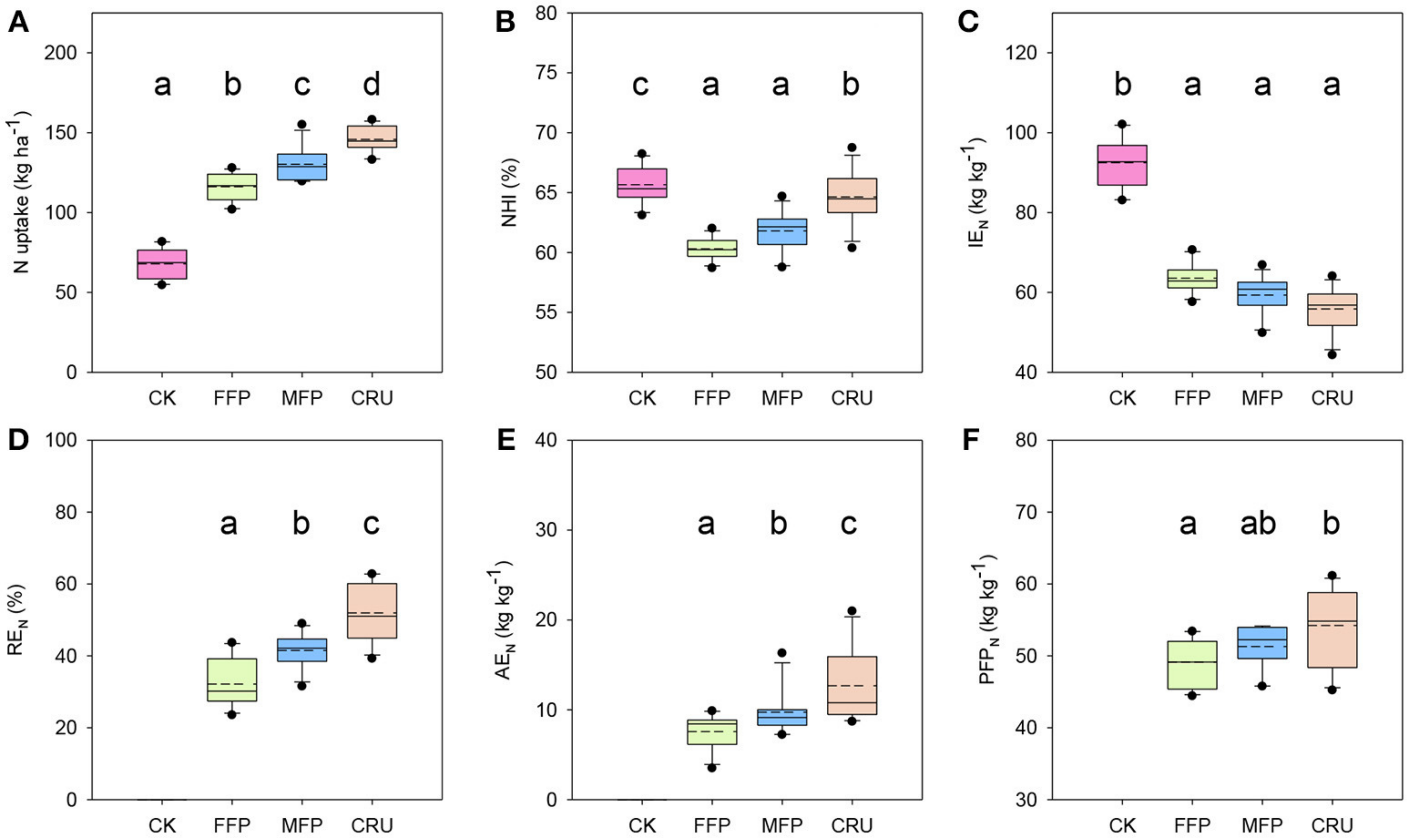
Comparison of nitrogen uptake and use efficiency by different fertilization treatments. **(A)** N uptake; **(B)** NHI, N harvest index = N in grains/N uptake; **(C)** IEN, internal N efficiency = grain yield/N uptake; **(D)** RE_N_, N recovery efficiency = [plant N accumulation in the plot received N fertilizer (T_N_) – plant N accumulation in the zero-N control (T_0_)]/amount of N applied (N_A_); **(E)** AE_N_, N agronomic efficiency = [grain yield in the plot received N fertilizer (G_N_) – grain yield in the zero-N control (G_0_)]/N_A_; **(F)** PFP_N_: nitrogen partial factor productivity = G_N_/N_A_. Results are pooled from different years and locations. Boxes followed by the same letter are not significantly different at 5% level.

N treatment influenced rice N uptake and N use efficiencies across field experiments, as shown in Figure 1. Rice crops in CK plots had the lowest N uptake. N application significantly improved crop N uptake. Relative to CK, FFP significantly increased crop N uptake, by 71.0% on average. MFP further enhanced the average crop N uptake to a level 12.1% higher than for FFP. Crop N uptake was the highest for CRU, ranging from 134.7 to 158.1 kg ha^−1^ (Figure 1A).

The N harvest index (NHI) of CK reached 65.7% on average, the highest among N treatments (Figure 1B). CRU was the second highest with an average of 64.6%, significantly lower than for CK. However, the values were restricted to 60.3 and 61.8% for FFP and MFP, respectively, and were significantly lower than for the CRU treatment.

CK was the highest in internal N use efficiency (IE_N_) (Figure 1C). N application reduced rice IE_N_ substantially, but FFP, MFP, and CRU did not differ significantly, with mean values of 63.6, 59.3, and 55.9 kg kg^−1^N, respectively.

N recovery efficiency (RE_N_) varied significantly among N treatments, as shown in Figure 1D. FFP produced the lowest RE_N_, ranging from 23.5–43.7%. In contrast, the RE_N_ for MFP was significantly higher, with a mean of 41.5%. Moreover, the RE_N_ achieved for CRU was 51.9%, significantly higher than for FFP and MFP treatments.

The agronomic N use efficiency (AE_N_) for FFP was restricted to a level below 7.6 kg kg^−1^ N on average, as shown in Figure 1E. Relative to FFP, the mean AE_N_ for MFP was 28.8% higher. CRU further increased crop AE_N_ to a level that was 67.5 and 30.1% higher than FFP and MFP, respectively.

The N partial factor productivity (PFP_N_) for FFP was in a range between 44.4 and 53.4 kg kg^−1^ N (Figure 1F). The mean PFP_N_ for MFP was 51.3 kg kg^−1^ N, which did not differ significantly from that for FFP treatment. However, CRU further increased the mean PFP_N_ to 54.2 kg kg^−1^ N, a level 10.4% higher than for the FFP treatment.

### Container experiment

#### Nitrogen release of controlled-release urea

**Figure 2:**
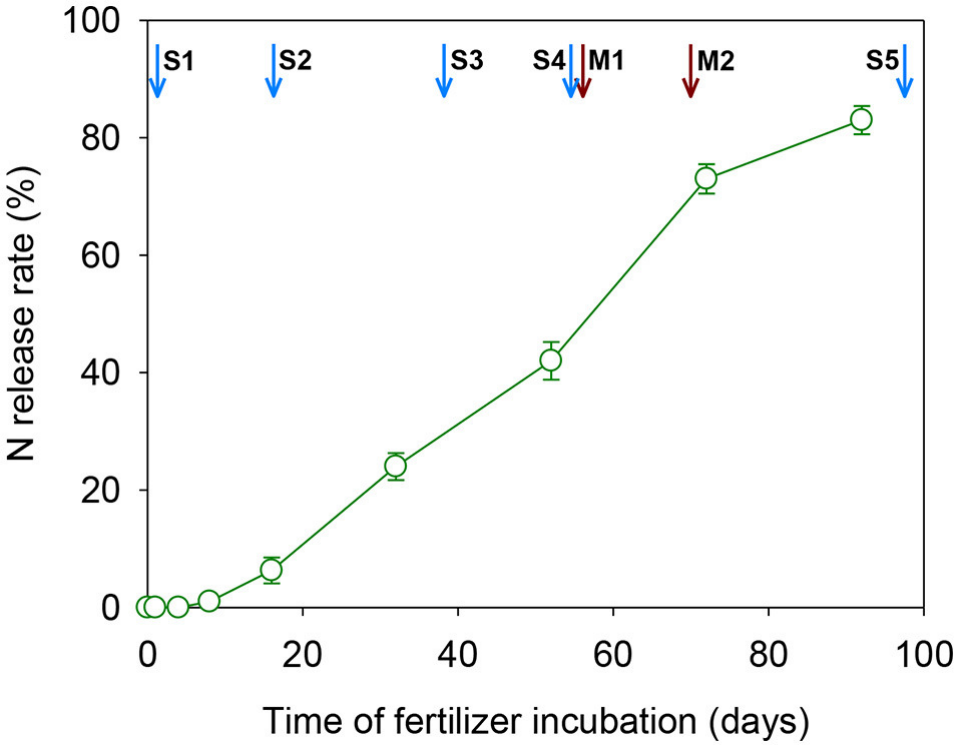
Nitrogen release curve of the CRU under the container experimental condition. Arrows in blue indicate the key rice growth stages: S1, transplanting; S2, tillering; S3, panicle initialing; S4, flowering; S5, maturity. Arrows in red indicate times of physiological measurements: M1, 3DAA; M2, 17DAA.

The N cumulative release rate of CRU followed a “S” shape curve under the experimental conditions, as shown in Figure 2. The N release of CRU initiated with a slow release stage at the first 16 days, then followed by an accelerated release stage (16–72 days), and finished with a reduced N release stage after 72 days. The N cumulative release rates reached 6.3, 73.2 and 82.9% respectively. These results indicated that the N release longevity of the CRU was about 90–100 days under the present experimental conditions.

### Dry matter, N accumulation, and translocation

In consistence with the results of field experiments, considerable yield variations were found among N treatments in the container experiment (Table 4). The grain yield achieved for CK was 18.8 g hill^−1^, the lowest of all the treatments. By contrast, FFP significantly increased grain yield by 35.2%. Relative to FFP, MFP further increased grain yield by 28.0%. The highest grain yield was produced by the CRU treatment, which was 9.9% higher than for MFP.

**Table 4:** Grain yield (GY), aboveground dry weight (DW) and N uptake at anthesis and physiological maturity, dry matter translocation efficiency (DMTE) and contribution to grain yield (CDMRG), nitrogen translocation efficiency (NTE) and contribution to grain N (CNRG) in the container experiment.

**Treatment**	**GY (g hill^−1^)**	**DM (g hill**^**−1**^**)**			**DMT (g hill^−1^)**	**DMTE (%)**	**CDMRG (%)**	**N uptake (g hill**^**−1**^**)**			**NT (g hill^−1^)**	**NTE (%)**	**CNRG (%)**
		**Anthesis**	**Maturity**	**Post-anthesis**				**Anthesis**	**Maturity**	**Post-anthesis**			
CK	18.8 a	25.5 a	37.8 a	12.3 a	6.7 a	26.3 b	35.6 b	0.24 a	0.30 a	0.06 a	0.14 a	57.2 b	72.5 c
FFP	25.4 b	36.4 b	54.2 b	17.8 b	8.3 b	22.7 a	32.9 ab	0.37 b	0.48 b	0.11 b	0.18 b	50.0 a	62.8 b
MFP	32.5 c	40.3 c	63.4 c	23.0 c	9.0 b	22.2 a	27.5 a	0.47 c	0.63 c	0.16 c	0.22 c	47.1 a	56.9 a
CRU	35.7 d	43.5 c	69.6 d	26.2 d	10.1 c	23.2 a	28.3 a	0.51 c	0.68 c	0.17 c	0.24 c	46.5 a	54.4 a

*Within a column, means followed by the same letters are not significantly different at 5% level. GY = 0.75 DMPost-anthesis + 2.21 DMT – 5.62, R^2^ = 0.985, P < 0.001*.

Crop DM varied with N treatments, as shown in Table 4. Crop DM was the highest for CRU, then MFP and FFP, with the lowest for CK at both anthesis and physiological maturity. In addition, CK crops accumulated 12.3 g hill^−1^ DM from anthesis to maturity, which was also the lowest for the four treatments. By contrast, N application treatments significantly increased post-anthesis DM accumulation, increasing for FFP and MFP by 44.7 and 87.0%, respectively (*P* < 0.05). CRU application further improved post-anthesis DM accumulation to a value 47.2 and 13.9% higher than in FFP and MFP, respectively (*P* < 0.05).

The DM translocated post-anthesis (DMT) in CK was the lowest for the four treatments. Comparable values of DMT were observed for FFP and MFP, which were significantly higher than for CK. CRU enhanced crop DMT the most, which was 10.1 hill^−1^, significantly higher than for the other three treatments. However, N application significantly lowered the efficiency of DM translocation (DMTE) compared to CK. CRU, MFP, and FFP did not differ significantly in DMTE.

For CK crops, the DM translocation after anthesis contributed (CDMRG) 35.6% to grain yield, the highest for the four treatments. The CDMRG for FFP was comparable with that for CK. MFP and CRU reduced CMDRG to 27.5 and 28.3%, respectively, significantly lower than for CK.

The amount of N uptake was the lowest for CK at both anthesis and maturity (Table 4). In contrast, it was significantly improved for N application treatments, among which CRU had a crop N uptake of 0.51 and 0.68 g hill^−1^ at anthesis and maturity, slightly (not statistically significantly) higher than for MFP, while FFP was the lowest in crop N uptake. Accordingly, crop N uptake post-anthesis was the highest for CRU and MFP, followed by FFP, with the lowest being for CK.

The amount of N translocated post-anthesis (NT) for CK was the lowest of the four treatments. Relative to CK, FFP increased NT by 28.6%. CRU and MFP further improved NT to 0.24 and 0.22 g hill^−1^, 33.2 and 22.2% higher than for FFP, respectively. However, N application lowered the efficiency of N translocation (NTE) in comparison to CK. Treatments with N applications did not differ significantly in NTE.

N translocation contributed (CDMRG) 72.5% of grain N for CK crops, the highest of the four treatments (Table 4). FFP produced the second highest CNRG at 62.8%. The CNRG values for MFP and CRU were comparable (56.9 and 54.4%, respectively), but lower than for FFP (62.8%).

### Flag leaf SPAD, photosynthesis, root oxidation activity, soil ${\text{NH}}_{4}^{+}$-N content during grain filling

**Figure 3:**
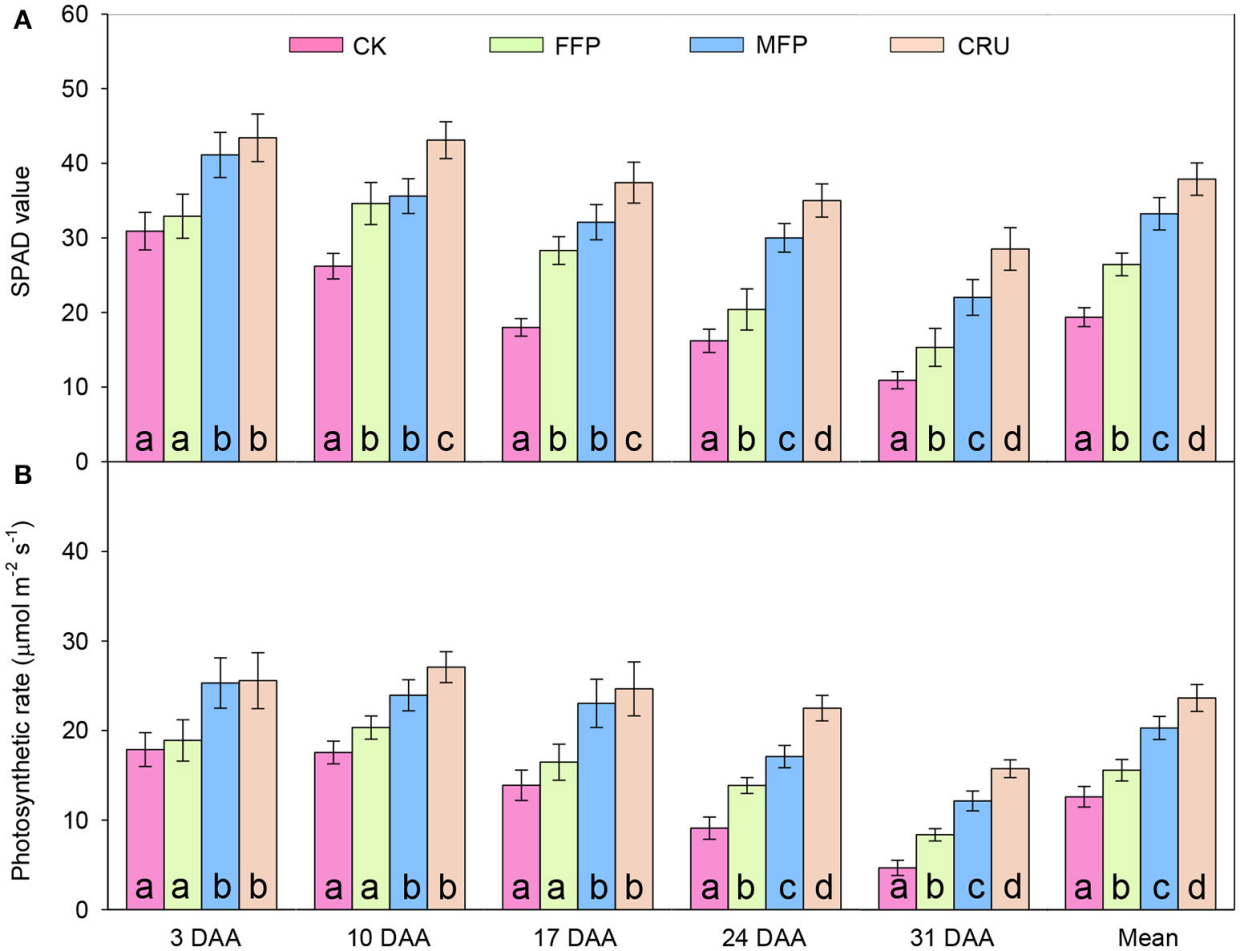
SPAD value **(A)** and photosynthetic rates **(B)** of flag leaves in different fertilizer treatments in the container experiment. DAA, days after anthesis. Bars (mean ± SD) followed by the same letter are not significantly different at 5% level.

Flag leaf SPAD decreased gradually with days after anthesis (DAA). N application improved flag leaf SPAD after anthesis (Figure 3A). CRU always had the highest measured SPAD values, while CK had the lowest overall. At 3 DAA, the flag leaf SPAD for four treatments had two distinct levels, i.e., MPF and CRU had comparable SPADs, which were significantly higher than for the CK and FFP treatments. At 10 and 17 DAA, CRU was the highest in flag leaf SPAD, followed by MFP and FFP, with CK being the lowest. At 24 and 31 DAA, CRU still had the highest flag leaf SPAD, but the values reduced to 35.0 and 28.5, respectively. MFP had the second highest flag leaf SPAD, followed by FFP, with the lowest for CK. Averaged across measurements, flag leaf SPAD appeared to have the following trend: CRU > MFP > FFP > CK.

At the early stages after anthesis (3, 10, and 17 DAA), the photosynthetic rates for the four treatments separated into two distinct levels (Figure 3B): CRU and MFP treatments were comparable, but were significantly higher than those for FFP and CK. At the late stages (24 and 31 DAA), photosynthetic rates differed significantly between treatments and could be ranked in a clear order: CRU > MFP > FFP > CK. Averaged over all measurements, the mean photosynthetic rate attained for CRU was 23.7 μmol m^−2^ s^−1^, the highest of the four treatments. MFP was the second highest (20.3 μmol m^−2^ s^−1^), followed by FFP (15.6 μmol m^−2^ s^−1^), while CK was the lowest (12.6 μmol m^−2^ s^−1^).

**Figure 4:**
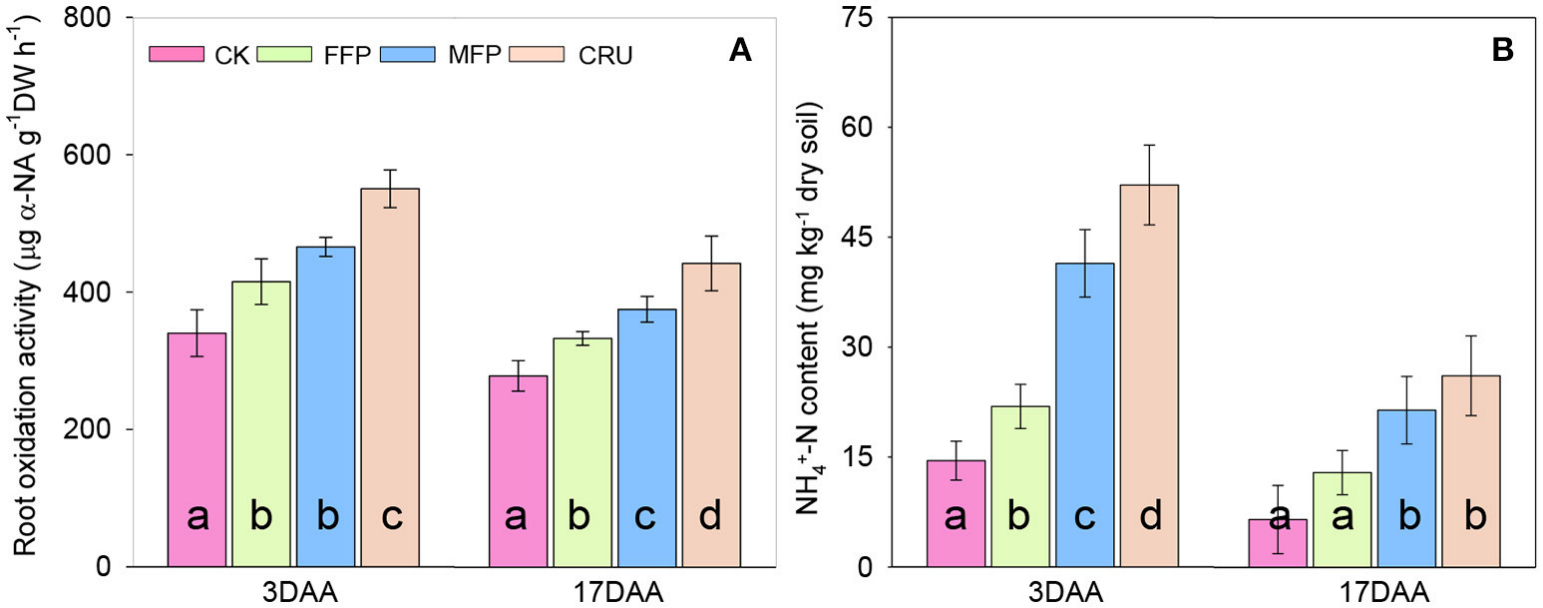
Root oxidation activity **(A)** and soil NH4+-N content **(B)** during grain filling. DAA, days after anthesis. Bars (mean ± SD) followed by the same letter are not significantly different at 5% level.

In agreement with the improved SPAD and photosynthetic rates of flag leaf, crops having N applications also retained higher root oxidation activity after anthesis (ROA, Figure 4A). At 3 DAA, ROA was improved the most for CRU, followed by MFP and FFP, while the lowest was found for CK. With the process of grain filling, ROA decreased significantly. At 17 DAA, CRU was still the highest in ROA. MFP had the second highest ROA, followed by FFP, with CK having the lowest value.

After anthesis, soil N was mainly in the form of ${\text{NH}}_{4}^{+}$-N and only ${\text{NH}}_{4}^{+}$-N content was significantly affected by N fertilization patterns in paddy soil. Therefore, only ${\text{NH}}_{4}^{+}$-N content was shown (Figure 4B). At 3 DAA, soil ${\text{NH}}_{4}^{+}$-N content was the highest in CRU. The second highest ${\text{NH}}_{4}^{+}$-N content occurred in MFP, followed by FFP, while the lowest was in CK. Soil ${\text{NH}}_{4}^{+}$-N content decreased as time proceeded and halved at 17DAA compared to that at 3DAA. At 17DAA, the soil ${\text{NH}}_{4}^{+}$-N contents in CRU and MFP were significantly higher than in FFP and CK.

### Activities of GS, GOGAT, and NR of flag leaf during grain filling

**Figure 5:**
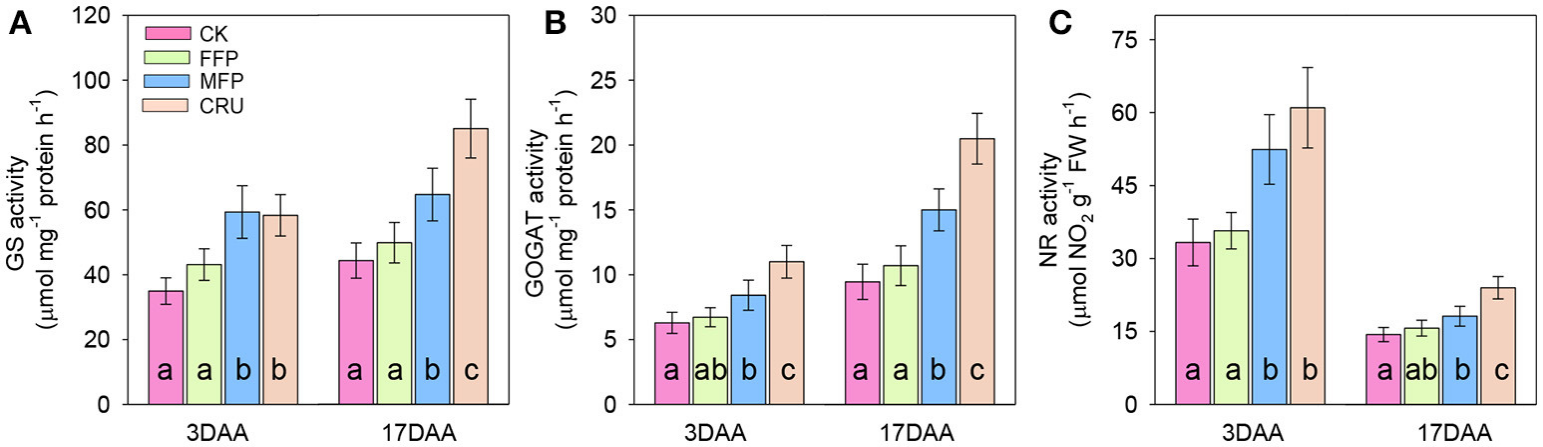
GS **(A)**, GOGAT **(B)**, and NR **(C)** activities in flag leaves during grain filling. DAA, days after anthesis. Bars (mean ± SD) followed by the same letter are not significantly different at 5% level.

The activities of N metabolism enzymes in flag leaf, including GS, GOGAT, and NR were illustrated in Figure 5. N treatments significantly influenced the activities of such main N assimilatory enzymes. At 3DAA, CRU, and MFP had similar levels of flag leaf GS activity, which were significantly higher than that in FFP and CK treatments. At 17 DAA, the GS activity reached the highest in CRU, then followed by MFP, while the lowest were in FFP and CK.

At 3DAA and 17DAA, the flag leaf GOGAT activities in CRU treatment were significantly higher than in MFP, while the lowest were in FFP and CK.

Similarly, the flag leaf NR activities in CRU and MFP treatments were significantly higher than in FFP and CK at 3DAA. The NR activity in CRU was still the highest at 17DAA. MFP was the second highest, followed by FFP, while CK was the lowest.

## Discussion

### N fertilizer, rice yield, and N use efficiency

The high rates of N fertilizer input and incorrect timing of N application were two important factors that resulted in low N use efficiencies in the rice cropping system in China (Peng et al., 2010). One reason for excessive N application when using the farmers' local practice method might be lack of knowledge of the indigenous soil's N supply capacity which is much higher in irrigated rice fields in China than in other major rice growing countries (Peng et al., 2010). In this study, the average grain yield of the N-omission plots was 6.24 Mg ha^−1^ (Table 3), indicating a large indigenous N supply (Janssen et al., 1990). The grain yield of the treatments receiving 150 kg ha^−1^ N averaged approximately 7.76 Mg ha^−1^, similar to that achieved with the treatments using 180 kg ha^−1^ or more N (7.91 Mg ha^−1^, unpublished data). These results were consistent with previous reports (Qiao et al., 2012; Yao et al., 2012; Wu et al., 2013), suggesting that a moderate N application, i.e., 150 kg N ha^−1^ in our field experiments, produced the optimum yield potential; further increases in N supply did not increase grain yield.

Apart from an appropriate N amount, a rational distribution across different growing stages was necessary for high grain yield and efficient use of N (Zeng et al., 2012), because the synchronization between N supply and crop demand is the key to optimizing tradeoffs among yield, N efficiency, and environmental protection in crop production (Sui et al., 2013). Previous studies have indicated low N use efficiency in farmers' fertilizer practices, in which most N, if not all, was applied during the early vegetative stages (Zeng et al., 2012; Chen et al., 2015), leading to poor synchronization between N supply and crop demand. This was further confirmed in the present study, as FFP had the lowest RE_N_ and AE_N_ values of all the treatments receiving N fertilizer. Moreover, it is worth noting that basal application of urea in this study corresponded with the plum rain period (the East Asian rainy season) (Guo et al., 2017). The heavy rainfall and high temperature could lead to severe N losses even with a moderate N rate (Li et al., 2017, 2018), which may also contribute to the low N use efficiency in FFP.

Several N application strategies and CRU products (Yang et al., 2012; Ye et al., 2013; Ke et al., 2017; Li et al., 2017) have been suggested for improving rice yield and N use efficiencies while reducing N losses. However, there were inconsistent results for the beneficial effects of CRU in enhancing rice yield (or AE_N_) and apparent N recovery. In accordance with previous reports (Zeng et al., 2012; Chen et al., 2015), we found that urea split application in MFP increased rice RE_N_ and AE_N_ by 29.2 and 28.8%, respectively when compared to FFP (Figures 1D,E). In addition, a further comparison revealed that rice RE_N_ and AE_N_ were enhanced by 25.1 and 30.1% using CRU over with urea split application. Similar results were reported by Kondo et al. (2005), who found substantially higher improvements in RE_N_ and rice yield with a single basal application of CRU than with urea split applications. A higher rice N recovery with CRU was also proved in the field experiments carried out in Northeast Thailand (Ohnishi et al., 1999). In their experiments, the fertilizer RE_N_ was 71.2% with application of CRU150, thus much higher than with frequent split urea application (55.5%). However, they did not detect a significant difference in rice AE_N_. Golden et al. (2009) found the rice yield and RE_N_ for CRUs were always lower than for urea at the same N rates in a direct-seeded, delayed-flood rice system. Therefore, the outcome of using CRU is dependent on the environmental conditions in a given region, which may result in variation in N release characteristics and the synchronization with crop demand. The findings from our experiments clearly suggested that rice yield and N efficiencies using CRU application could surpass those from using urea split application, while reducing the number of topdressing applications and saving labor to achieve better economic and environmental benefits.

### Yield components

**Figure 6:**
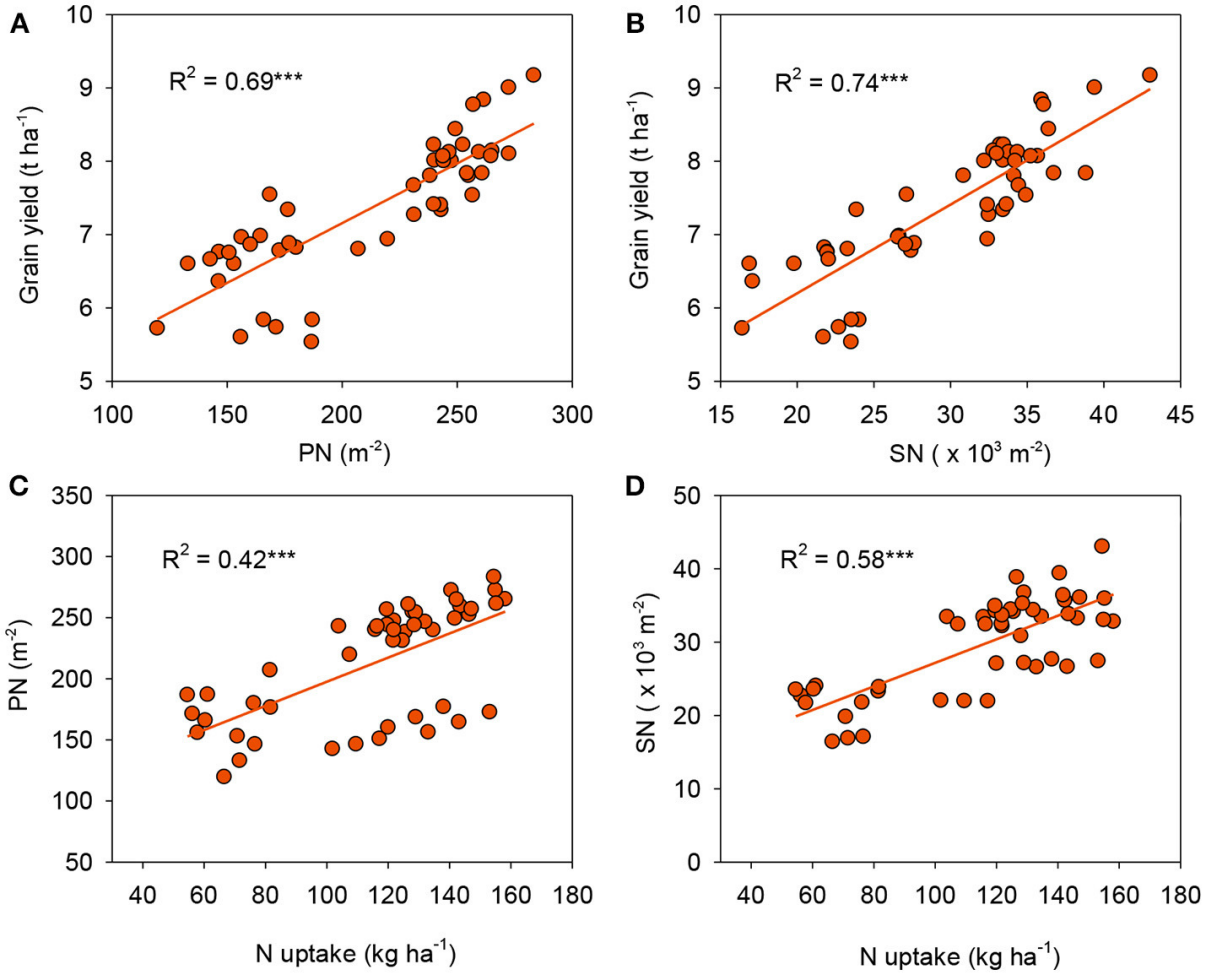
Linear relationships of grain yield with panicle number [PN, **(A)**] and spikelet number [SN, **(B)**], and PN **(C)** and SN **(D)** with *N* uptake. ^***^p < 0.001.

Rice yield is determined by yield components such as PN, SPP, SN, and GW. In agreement with findings of previous work (Sui et al., 2013), our results demonstrated that PN and SN were most variable among the components detected (Table 3). The highest rice yield for CRU might be explained by the enhanced PN and SN, while there were no negative effects on GW. This was further supported by the significant and positive relationships between grain yield with both PN (*R*^2^ = 0.69, *P* < 0.001, Figure 6A) and SN (*R*^2^ = 0.74, *P* < 0.001, Figure 6B). PN has been considered to be the main factor determining yield because the effect of increasing PN was similar to that of increasing rice yield (Lee et al., 2010). Moreover, our results further demonstrated that PN and SN were positively correlated with N uptake (*R*^2^ = 0.42, *P* < 0.001 and *R*^2^ = 0.58, *P* < 0.001, respectively, Figures 6C,D), suggesting that a greater N uptake enhanced the formation of a larger sink (as quantified by SN), and therefore led to greater rice yield. Similar results were reported by Chen et al. (2015).

Increased N application at later growth stages has been reported to increase SPP (Sun et al., 2012). In this study, the SPP was slightly lower in N-omission plots (only significant in Honghu 2010) than the treatments with N application. However, no significant difference existed among three N application practices that might result in variations in soil N availability at later growth stages. This phenomenon might be ascribed to the fact that the competition for nutrients and carbohydrates became more and more intense with the increase of PN in MFP and CRU over FFP, which may have led to more spikelet degeneration and thus lessened the effects of higher number of differentiated spikelets per panicle. Similar results were also reported by Sui et al. (2013). Furthermore, we did not find N management effects on GW, which, in agreement with previous reports (Zeng et al., 2012; Sui et al., 2013), indicated it was a rather stable varietal character that was mostly determined by its genotype.

### Post-anthesis DM accumulation and translocation

From another perspective, grain yield was the function of post-anthesis biomass production and translocation of biomass accumulated before flowering to grains (Yang et al., 2008; Wu et al., 2013; Pal et al., 2017). Previous studies have emphasized the importance of the former or the latter processes for increased rice yield. Pal et al. (2017) determined that the contribution of pre-anthesis DM to grain varied from 20.7 to 67.9%, depending on rice genotypes and sowing dates. In their investigation, the increased grain yield in the early planted crop was essentially due to a higher crop DM at anthesis that allowed a higher remobilization of resources to the developing grains. However, Wu et al. (2013) reported the yield variation between the early and late season rice crops could be explained mostly by the differences in biomass accumulation after flowering. In this study, CRU had both the highest DMT and post-anthesis DM accumulation of all four treatments, but post-anthesis accumulation was a major contributor to grain yield (64.4–72.5%, Table 4). Furthermore, stepwise multiple regression analysis showed that post-anthesis DM accumulation accounted for 65.9% of yield variation, while DMT explained 32.6%. Such findings further highlighted that both these processes were crucial, but post-anthesis DM accumulation was more important in determining grain yield in this study.

In support of greater post-anthesis DM accumulation, CRU had higher leaf SPAD and photosynthetic rates than MFP and FFP during the grain filling period (Figures 3A,B). These results indicated that CRU delayed leaf senescence and thus led to higher photosynthetic activity and prolonged time. Furthermore, apart from the higher NT, CRU and MFP had a significantly increased post-anthesis N uptake, compared to FFP and CK, while there was the opposite trend in the variation of CNRG (Table 4). It seems likely that, with increased soil available N at anthesis (Figure 4B), rice crops being given CRU treatment relied more on post-anthesis N uptake for grain N filling, which could allow and compensate for the slower leaf senescence and N remobilization. In corroboration, significantly higher root oxidation activities were detected for CRU during the grain filling period (Figure 4A). These observations thus highlighted the role of N uptake after flowering in enhancing rice yield. However, the regulation of carbon (C) and N assimilation post-anthesis was closely correlated. In rapeseed and maize, enhanced N uptake after flowering was accompanied by delayed leaf senescence (Mi et al., 2003; Erley et al., 2007). A delayed leaf senescence can lead to a better assimilation supply to the roots, so that high root activity and N uptake is maintained and, in turn, leaves have a longer lifespan (Osaki, 1995). Some recent research has also suggested that enhanced root activity after anthesis was involved in synthesis of plant hormones, especially cytokinin, which would be translocated to the uppermost leaves, helping to regulate photosynthetic acclimation (Gu et al., 2017).

GS, GOGAT, and NR are the key enzymes involved in N assimilation of plants (Tabuchi et al., 2007; Sun et al., 2015). In consistent with the increased soil N availability and crop N uptake after anthesis (Figure 4, Table 4), CRU treatment generally improved GS, GOGAT, and NR activities in rice flag leaves (Figure 5). These results indicated that, CRU treatment increased N assimilation in functional leaves more than the conventionally applied urea, which was essential for providing sufficient substrate for grain filling and thus contributing to higher grain yield (Sun et al., 2012). In support of our results, Yang et al. (2012) reported close relationships between soil N availability and cumulative N uptake with the activities of key N assimilatory enzymes (GS, GOGAT, and NR) of rice leaves during grain filling stage. Sun et al. (2012) has established that, rice yield was positively and significantly correlated with the activities of GS and GOGAT in function leaves at heading and maturity stages. Moreover, significant and positive correlations have previously been observed between root activity with N assimilation enzymes and photosynthetic rate (Sun et al., 2012). It could be confirmed in the present study, as the ranks of these indexes in different N treatments generally followed similar patterns (i.e., CRU > MFP > FFP > CK). These results indicated that, the underground processes were closely linked to that aboveground during grain filling, which together contributed the high rice yield under CRU treatment.

## Conclusion

Significantly higher rice grain yields and N efficiencies were achieved with a single basal application of CRU under the existing field conditions, as compared to conventional urea applied either as basal or split topdressing. The higher grain yield with CRU was mainly due to improved N uptake that resulted in greater panicle and spikelet numbers per m^2^. The finding in our study highlighted the importance of post-anthesis DM production for high yield in irrigated rice systems. Further physiological studies revealed CRU increased post-anthesis N uptake of rice crops through sustainable N release, which could maintain higher photosynthetic rates and compensate for the slower leaf senescence. Moreover, CRU crops were considerably higher root activity, and also had higher activities in N assimilatory enzymes (GS, GOGAT, and NR) in flag leaves during grain filling stage. Taken together, these underground and aboveground processes have contributed to the higher grain yield with CRU application.

## Author contributions

LW, FC, and YL initiated and designed the research. LW, CX and YL performed the experiments and collected the data. XP measured the key enzymes of N assimilation. LW analyzed the data and wrote the manuscript. FC and YL edited the manuscript and provided guidance during experimentation.

### Conflict of interest statement

The authors declare that the research was conducted in the absence of any commercial or financial relationships that could be construed as a potential conflict of interest.
